# Estimating Daytime Ecosystem Respiration to Improve Estimates of Gross Primary Production of a Temperate Forest

**DOI:** 10.1371/journal.pone.0113512

**Published:** 2014-11-24

**Authors:** Jinwei Sun, Jiabing Wu, Dexin Guan, Fuqi Yao, Fenghui Yuan, Anzhi Wang, Changjie Jin

**Affiliations:** 1 State Key Laboratory of Forest and Soil Ecology, Institute of Applied Ecology, Chinese Academy of Sciences, Shenyang, P.R. China; 2 Changjiang River Scientific Research Institute, Wuhan, P.R. China; Universidade Federal de Vicosa, Brazil

## Abstract

Leaf respiration is an important component of carbon exchange in terrestrial ecosystems, and estimates of leaf respiration directly affect the accuracy of ecosystem carbon budgets. Leaf respiration is inhibited by light; therefore, gross primary production (GPP) will be overestimated if the reduction in leaf respiration by light is ignored. However, few studies have quantified GPP overestimation with respect to the degree of light inhibition in forest ecosystems. To determine the effect of light inhibition of leaf respiration on GPP estimation, we assessed the variation in leaf respiration of seedlings of the dominant tree species in an old mixed temperate forest with different photosynthetically active radiation levels using the Laisk method. Canopy respiration was estimated by combining the effect of light inhibition on leaf respiration of these species with within-canopy radiation. Leaf respiration decreased exponentially with an increase in light intensity. Canopy respiration and GPP were overestimated by approximately 20.4% and 4.6%, respectively, when leaf respiration reduction in light was ignored compared with the values obtained when light inhibition of leaf respiration was considered. This study indicates that accurate estimates of daytime ecosystem respiration are needed for the accurate evaluation of carbon budgets in temperate forests. In addition, this study provides a valuable approach to accurately estimate GPP by considering leaf respiration reduction in light in other ecosystems.

## Introduction

Leaf respiration can account for up to 25% or higher proportions of daily photosynthesis [1]–[3] and therefore plays an important role in the estimation of gross primary production (GPP). In previous studies, leaf respiration in light is considered to be the same magnitude as respiration at night [4], while further studies found that leaf respiration is inhibited in light. The extent of light inhibition of leaf respiration ranges from 25% to 100% [5]–[8]. Traditionally, ecosystem respiration (*R*_e_) was estimated using simple functional relationships associated with temperature and soil water availability [9]–[12]. However, this method is limited because inhibition of leaf respiration in light (*R*_L_) is not considered [5],[13]–[14]. Therefore, *R*_e_ and GPP are overestimated when daytime *R*_e_ is estimated from nighttime *R*_e_ [15]–[17]. Accounting for leaf respiration inhibition in the daytime could therefore markedly improve the accuracy of ecosystem GPP estimation [8],[17]–[18].

Although previous studies investigated the difference between *R*_L_ and leaf respiration in darkness (*R*_D_), to our knowledge, few researchers have incorporated this difference into ecosystem-level studies. Tingey *et al.* (2007) reported that daytime *R*_e_ values of Douglas fir seedlings were 28% and 16% lower under ambient and elevated CO_2_ concentrations, respectively, compared with the values obtained when light inhibition of leaf respiration was ignored [19]. In an old beech forest, GPP calculated after taking light inhibition of *R*_e_ into account was only 76% of the value obtained when light inhibition was not considered [16]. Although the above studies estimated *R*_e_ or GPP more accurately by applying the daytime leaf respiration reduction to the canopy level [16],[19], their estimates did not consider the attenuation of radiation in the canopy. Additionally, in a study conducted by Bruhn *et al.* (2011), the low photosynthetically active radiation (*Q*) levels used in the Kok method were frequently acquired in early morning or at nightfall and were very limited in number after data screening [16]. This limitation occurred because friction velocity (*u* ^*^) is generally low at those times, while the data screening standard is *u* ^*^>0.5. Some reports state that the degree of light inhibition varies with *Q* [17].[20]–[21], but failure to consider *Q* differences in the canopy leads to an error in canopy respiration estimation and consequently, the evaluation of *R*_e_ and GPP. Radiation in a layered canopy should be estimated when the daytime inhibition at the leaf and canopy level needs to be assessed because radiation is attenuated with increasing canopy depth. Wohlfahrt *et al.* (2005) divided the canopy of a mountain meadow into a statistically sufficient number of layers and produced a more accurate calculation of GPP at the canopy level, showing that GPP declined by 11% to 13% (using a low estimate of leaf respiration inhibition), and by 13% to 17% (using a high estimate of leaf respiration inhibition) [17]. In this study, the estimate of dark respiration was limited because daytime inhibition of leaf-level respiration was provided as a range of values based on the scattered results of several published studies reviewed by Wohlfahrt *et al.* (2005) [17]. There are no studies in forest ecosystems that have provided estimates as detailed as those of Wohlfahrt *et al.* (2005) [17].

In our study, we estimated leaf dark respiration using the Laisk method [22] and analyzed the *R*_L_ values under different *Q* levels for dominant tree species of a mixed temperate forest. Measurements were conducted on potted seedlings of dominant tree species to determine the effect of light inhibition on dark respiration of individual species. The reduction in *R*_e_ was estimated by separating the canopy into 20 layers to calculate GPP based on eddy covariance measurements and a multilayer model. The specific aims of the present study were to: (1) assess the leaf respiration reduction in light of specific species under different *Q* levels, (2) evaluate spatial differences in *Q* using a radiative transfer model and estimate leaf respiration reduction in light relative to darkness at the canopy level, and (3) evaluate the impacts of leaf respiration reduction in light on canopy respiration, *R*_e_, and GPP.

## Materials and Methods

### 2.1 Ethics Statement

The field studies were conducted at the Changbai Mountains National Nature Reserve in northeastern China. This is a practice base for the researchers of the Chinese Academy of Sciences. The experiments conducted in this study did not involve any protected animals or plants, and this study was permitted by the Station of Changbai Mountain Forest Ecosystem, Chinese Academy of Science.

### 2.2 Site description and materials

The National Natural Conservation Park of Changbai Mountain (42°24′N, 128°06′E; 738 m elevation) is situated in the temperate continental climatic zone [23]. The annual mean air temperature is 3.6°C, and mean precipitation is 695.3 mm according to meteorological records from 1982 to 2003. The soil is classified as dark brown forest soil. The growing season extends from May to September. The dominant tree species of this ecosystem are *Pinus koraiensis*, *Tilia amurensis*, *Fraxinus mandshurica*, *Acer mono*, and *Quercus mongolica*.

The experiment was performed in openings in a temperate broad-leaved Korean pine mixed forest, and four dominant tree species (*T. amurensis*, *F. mandshurica*, *A. mono*, and *P. koraiensis*) were selected. Four potted seedlings were selected for each tree species. For sampling purposes, four fully expanded and healthy representative leaves were randomly selected from each seedling and all measurements were averaged. The four mean values of each tree species were used as replicates for statistical analysis.

### 2.3 Measurement and estimation of leaf respiration

Leaf respiration of mature trees could not be measured directly considered the height of the trees, and therefore leaf respiration of seedlings of the dominant tree species was measured. Leaf respiration in light was estimated using the Laisk method by evaluating the net photosynthetic rate (*A*_n_) at a series of low intercellular CO_2_ concentrations (*c*_i_) under different *Q* levels. Photosynthetic measurements were conducted using an open-mode portable photosynthesis system (LI-6400, LI-COR, Lincoln, NE, USA), and *A*_n_ was calculated on an area basis. To determine the effect of light intensity on daytime respiration, *A*_n_ was measured at seven *Q* levels (50, 100, 150, 210, 300, 600, and 800 µmol·m^−2^·s^−1^) and CO_2_ concentrations (150, 120, 90, 70, 60, 50, and 40 µmol·mol^−1^). Linear regressions of *A*_n_ versus *c*_i_ were performed for each *Q* level. The linear regressions crossed at a point under each pair of *Q* values, and the *A*_n_ coordinate of this point represented the *R*_L_ that corresponded to the average of these two *Q* values. Seedling *R*_D_ was measured following 20 min of dark acclimation. During all measurements, the leaf temperature was maintained at approximately 25°C, relative humidity at approximately 60%, and the flow rate was set at 500 µmol·s^−1^. The measurements were performed from 08:00 to 12:00 a.m. on sunny days.

### 2.4 Ecosystem CO_2_ flux measurements

#### 2.4.1 Instruments

The carbon dioxide flux over the forest site was continuously measured at a height of 40 m using the eddy-covariance (EC) technique. The flux system included a triaxial sonic anemometer (CSAT3, Campbell Inc., USA) and a fast-response open-path CO_2_/H_2_O infrared gas analyzer (LI-7500, LI-COR, USA). Soil temperature was measured using multilevel thermocouple probes, and sensors were placed at 5, 10, 20, 50, and 100 cm below the soil surface. Air temperature was measured using HMP-45C sensors located at 32 and 60 m and recorded using a data logger (CR5000, Campbell Inc., USA).

#### 2.4.2 Flux calculations and corrections

Fluxes were calculated online at 30-min intervals based on the time series of vertical velocity (w′) and CO_2_ concentration (*c*′) fluctuations according to Reynolds decomposition: 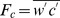. A coordinate rotation was applied to force the average vertical wind speed to zero and to align the horizontal wind to mean wind streamlines based on Wilczak *et al.* (2001) [24]. CO_2_ eddy fluxes were corrected for density effects using the WPL correction based on Webb *et al.* (1980) [25]. The underestimation caused by sensor line averaging, spatial separation, and high frequency losses was compensated by physically sound spectra correction following Moore (1986) [26].

Spikes and gaps in the archived records of EC measurements are inevitable because the sonic anemometer and infrared gas analyzer are sensitive to precipitation. In addition, power failures occur, and sensor calibration and maintenance are required. The data set coverage was approximately 78% for the above canopy EC system after testing for stationary and integral turbulence statistics using the method introduced by Foken and Wichura (1996) [27]. Linear interpolation was used to fill small gaps in data (<2 h) and the strategies proposed by Falge *et al.* (2001) [28] were used to fill large data gaps. When >30% of the data were missing for one day, only the total daily flux was calculated based on the relationship between CO_2_ flux processes and environmental variables [29].

### 2.5 Components of GPP

GPP is calculated by subtracting *R*_e_ from NEE. *R*_e_ includes mainly soil respiration (*R*_s_), stem respiration (*R*_st_), and canopy leaf respiration (*R*_c_). Soil, stem and canopy leaf respiration (ignoring the reduction in light) were calculated using an exponential function [30]:
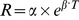
(1)where *R* is the respiration of the soil, stem, and canopy, and *T* is the temperature of these components. The parameters were obtained from the results presented in Wang *et al.* (2010) [30], who conducted research at this site (Table 1). There were small differences in stem respiration between the broad-leaved deciduous species (Table 1), and stem respiration of *A. mono* was used as the average value for the other three broad deciduous species. Light inhibition was not accounted for in the estimate of *R*_c_ when equation (1) was used. Therefore, the estimated *R*_c_ was higher than the true canopy leaf respiration in light and consequently, GPP was overestimated.

**Table 1:** Parameters of the temperature response in equation (2) for soil respiration and stem and leaf respiration of the dominant tree species (*P. koraiensis*, *T. amurensis*, *Q. mongolica*, and *F. mandshurica*) (µmol·m^−2^·s^−1^) (Wang *et al.*, 2010).

		*α*	*β*
Stem respiration	*Pinus koraiensis*	0.414	0.096
	*Tilia amurensis*	0.523	0.081
	*Quercus mongolia*	0.665	0.097
	*Fraxinus mandshurica*	0.408	0.114
Leaf respiration	*Pinus koraiensis*	0.250	0.035
	*Tilia amurensis*	0.232	0.042
	*Quercus mongolia*	0.238	0.087
	*Fraxinus mandshurica*	0.141	0.098
Soil respiration		0.640	0.101

### 2.6 Estimation of the reduction in canopy leaf respiration

**Figure 1:**
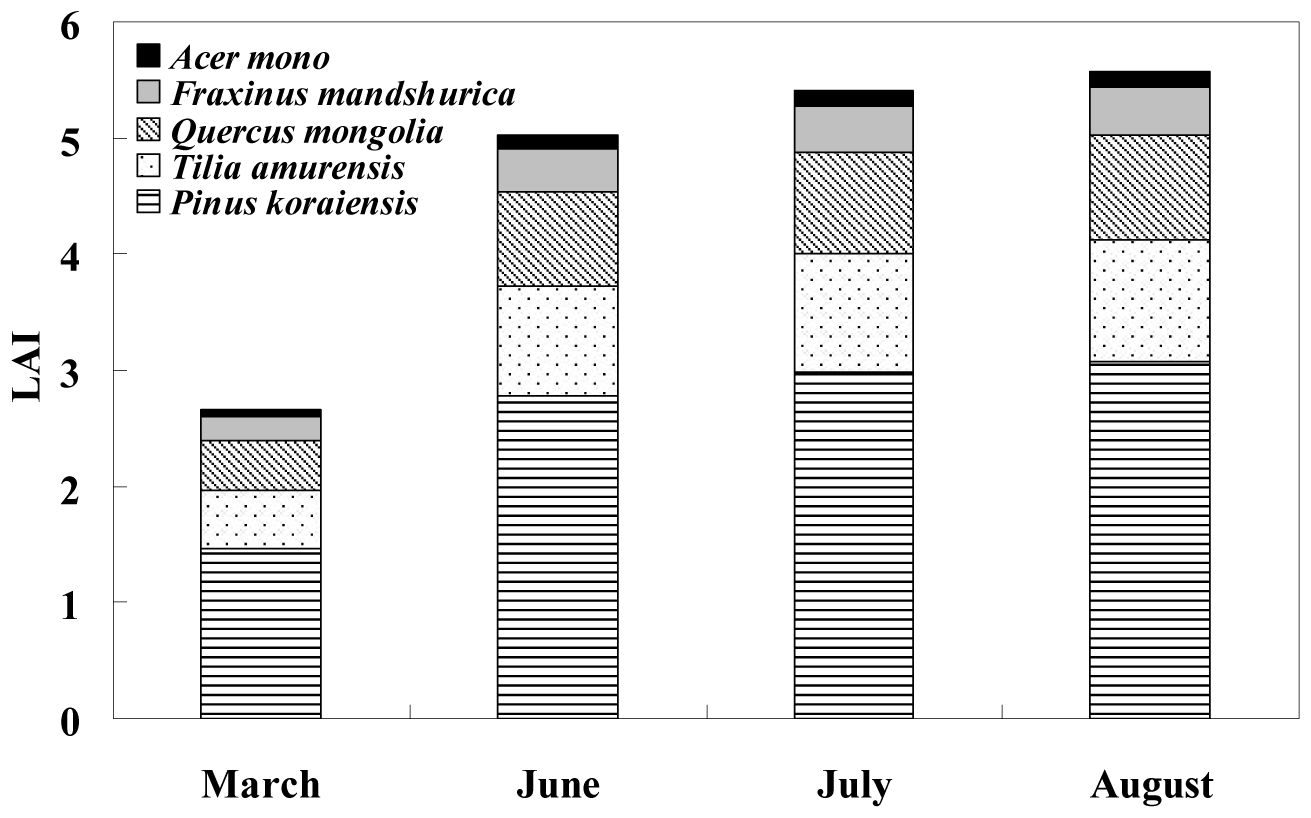
A typical case of mean canopy leaf area index (LAI) and the components of each tree species derived from the entire canopy during the growing season (May to September) in 2003.

The reduction in canopy leaf respiration in light relative to darkness (*F*) was estimated as follows:
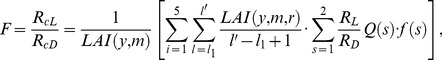
(2)where *m* (1 to 5) represents the month of the growing season from May to September, respectively, and *y* (1 to 3) represents the year from 2003 to 2005, respectively. LAI (*y*, *m*) is the canopy LAI determined by the month (*m*) and year (*y*), which is shown in Fig. 1. The values *i* = 1 to 5 represent the five dominant tree species, respectively; *l* represents the layer number from the top of the canopy for a tree species; *l*_1_ represents the first layer number, and *l*′ represents the last layer number; *l*′−*l*_1_+1 is the total number of layers per tree species; LAI (*y*, *m*, *r*) represents the LAI determined in a specific month and *r* represents the leaf biomass percent of a given species. The radiation absorption of a multilayer model [31] was used to estimate the *Q* in each canopy layer. Total canopy height ranged from 7 to 27 m based on field survey data collected near the flux tower. The range in tree height, leaf biomass, and the leaf biomass percent of the five dominant tree species are shown in Table 2 [32]. To apply the multilayer model to the mixed temperate forest, the canopy was divided into 20 layers. The thickness in each canopy layer was 1 m and the LAI was LAI/20. Sunlit and shaded leaves were treated separately for each canopy layer. Sunlit leaves receive both diffuse and direct beam radiation, whereas shaded leaves only receive diffuse radiation. The leaf angle distribution was assumed as a spherical distribution. Direct beam and diffuse radiation were calculated using the exponent profile method. The detailed radiation transmission model for the canopy is described in Appendix S1. The two values of *s* represent the cases of sunlit (*s* = 1) and shaded leaves (*s* = 2); *Q*(1) is the radiation received by sunlit leaves and *f*(1) is the fraction of sunlit leaf area; *Q*(2) and *f*(2) represent the radiation received by shaded leaves, and the fraction of the shaded leaf area, respectively. The ratio of canopy leaf respiration in light (*R*_cL_) to canopy leaf respiration in darkness (*R*_cD_) of sunlit and shaded leaves of each tree species was determined in each canopy sublayer, and *f_sl_* in each canopy layer is equal to the fraction of direct beams reaching that particular layer [33]:
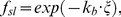
(3)where 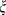 is the LAI accumulated from the top of the canopy and 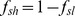.

**Table 2:** Tree height, canopy thickness and leaf biomass of five dominant tree species (*P. koraiensis*, *T. amurensis*, *Q. mongolica*, *F. mandshurica*, and *A. mono*) in the study forest (Shi *et al.*, 2010).

Species	Tree height range (m)	Leaf biomass (t×ha^−1^)	Percentage of leaf
*Pinus koraiensis*	9–22	3.13	55.3%
*Tilia amurensis*	9–22	1.07	18.9%
*Quercus mongolia*	9–25	0.91	16.1%
*Fraxinus mandshurica*	15–27	0.42	7.4%
*Acer mono*	7–17	0.13	2.3%

The canopy leaf respiration estimation assumes that temperature, humidity, wind velocity, and CO_2_ concentration are horizontally uniform in the canopy and that leaf biomass is uniformly distributed in the layers occupied by a given tree species. The mean discrepancy of air temperature between the top and bottom of the forest canopy was only 0.47°C based our measurements collected at the tower during the growing season from 2003 to 2005. The photosynthetically active radiation at the bottom of the canopy was attenuated by approximately 79%–94% compared with the top of the canopy under a leaf area index range of 2 to 6. Therefore, the temperature variation in the canopy was not considered. In the *R*_cL_/*R*_cD_ estimation, the *R*_L_/*R*_D_ values, calculated as a function of *Q* for the five dominant tree species, were obtained by measurements detailed in section 2.3, and the *R*_L_/*R*_D_ value of *Q. mongolica* was taken as the mean value of the other three broadleaved tree species.

### 2.7 Statistical analysis

Statistical analysis was performed using SPSS version 17.0 (SPSS, Chicago, IL, USA). Student's *t*-test was used to evaluate the differences between *R*_L_ and *R*_D_ for the dominant tree species. Relationships were fitted with polynomial functions that provided simple and well-fitting descriptions of the phenomena. All tests were based on a significance level of 0.05.

## Results

### 3.1 Light inhibition of leaf respiration

**Figure 2:**
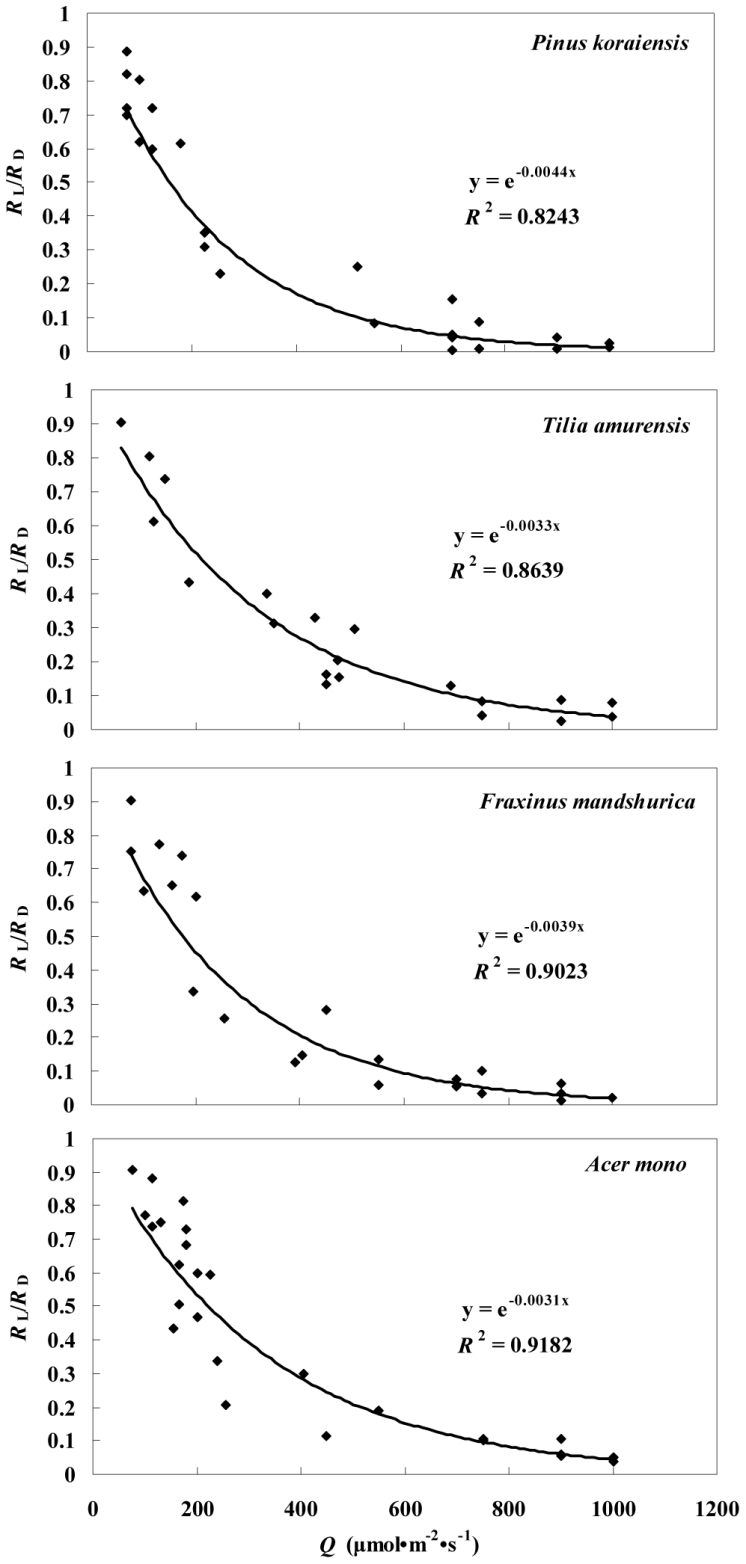
Variation in the ratio of *R*_L_ to *R*_D_ for four main tree species (*P. koraiensis*, *T. amurensis*, *F. mandshurica*, and *A. mono*) with different photosynthetically active radiation (*Q*) values. All relationships were significant at P<0.05.

The *R*_L_ values were lower (Student's *t*-test, P<0.05) than the *R*_D_ values for the dominant species. *R*_L_ declined exponentially with increasing *Q* levels, and the relationships between *R*_L_ and *Q* fit the following exponential equation:
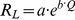
(4)These relationships were significant at P<0.05, and the values of *a* and *b* are listed in Table 3. Similarly, *R*_L_/*R*_D_ decreased exponentially with increasing *Q* levels and the variation in *R*_L_/*R*_D_ with *Q* is presented in Fig. 2 for the four dominant tree species.

**Table 3:** Parameter values of *a* and *b* in the functions listed in equation (5) for leaf respiration in light (*R*_L_), and photosynthetically active radiation (*Q*) of four dominant tree species (*P. koraiensis*, *T. amurensis*, *F. mandshurica*, and *A. mono*).

Species	*Pinus koraiensis*	*Tilia amurensis*	*Fraxinus mandshurica*	*Acer mono*
*a*	0.6672	0.7832	0.8808	0.8811
*b*	−0.0043	−0.0032	−0.0039	−0.0031

### 3.2 Estimation of canopy leaf respiration

**Figure 3:**
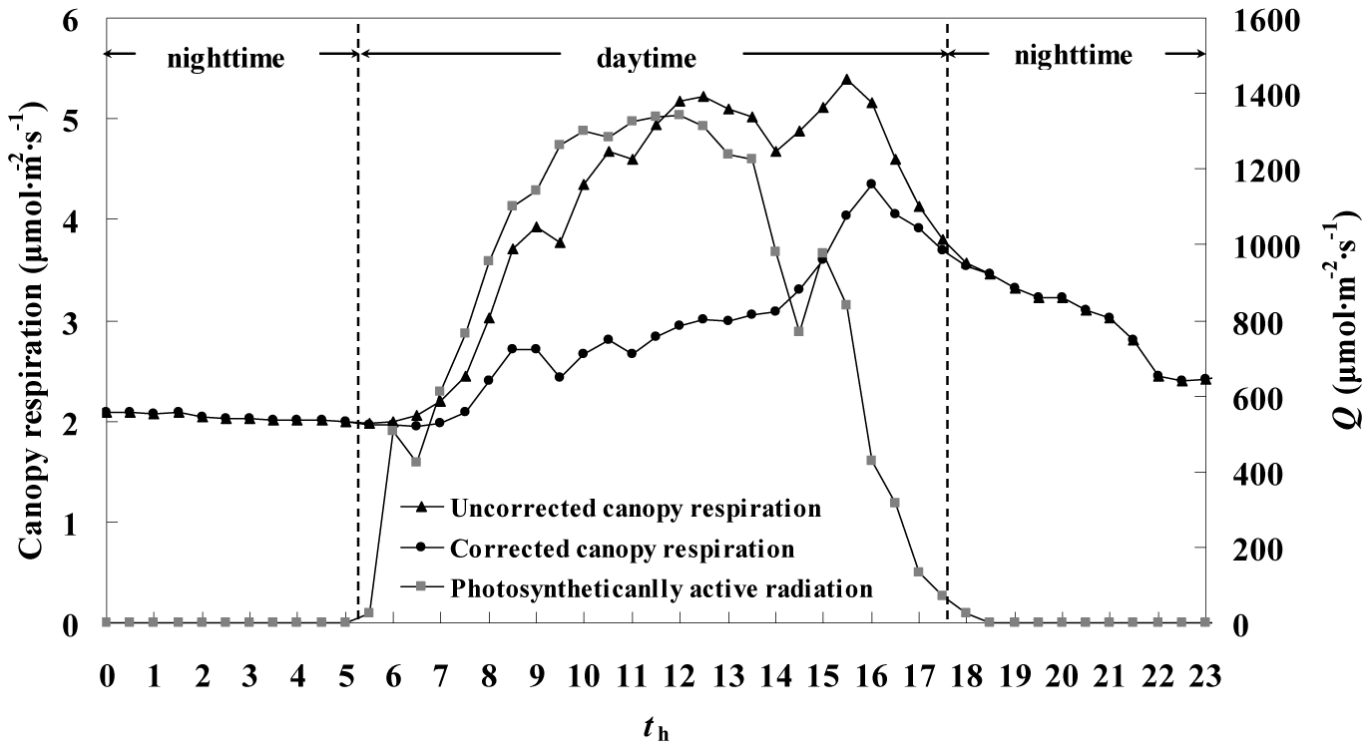
Diurnal variation of photosynthetically active radiation (*Q*) and canopy leaf respiration with and without daytime light inhibition correction.

Canopy leaf respiration without correction for light inhibition (*R*_c_) was estimated using equation (1), and the corrected canopy leaf respiration (*R*_c_^*^) was calculated by accounting for the reduction in leaf respiration during the daytime based on equation (2). The diurnal variation in *Q*, *R*_c_, and *R*_c_^*^ is shown in Fig. 3. At night, *R*_c_ was equal to *R*_c_^*^, whereas *R*_c_^*^ was lower than *R*_c_ in the day owing to light inhibition. The discrepancy between *R*_c_ and *R*_c_^*^ initially increased with *Q* and reached its highest value (2.23 µmol·m^−2^·s^−1^) at midday when *Q* was highest. The discrepancy decreased with decreasing *Q* during the afternoon. The diurnal variation is readily explained by equation (2) and (4).

**Figure 4:**
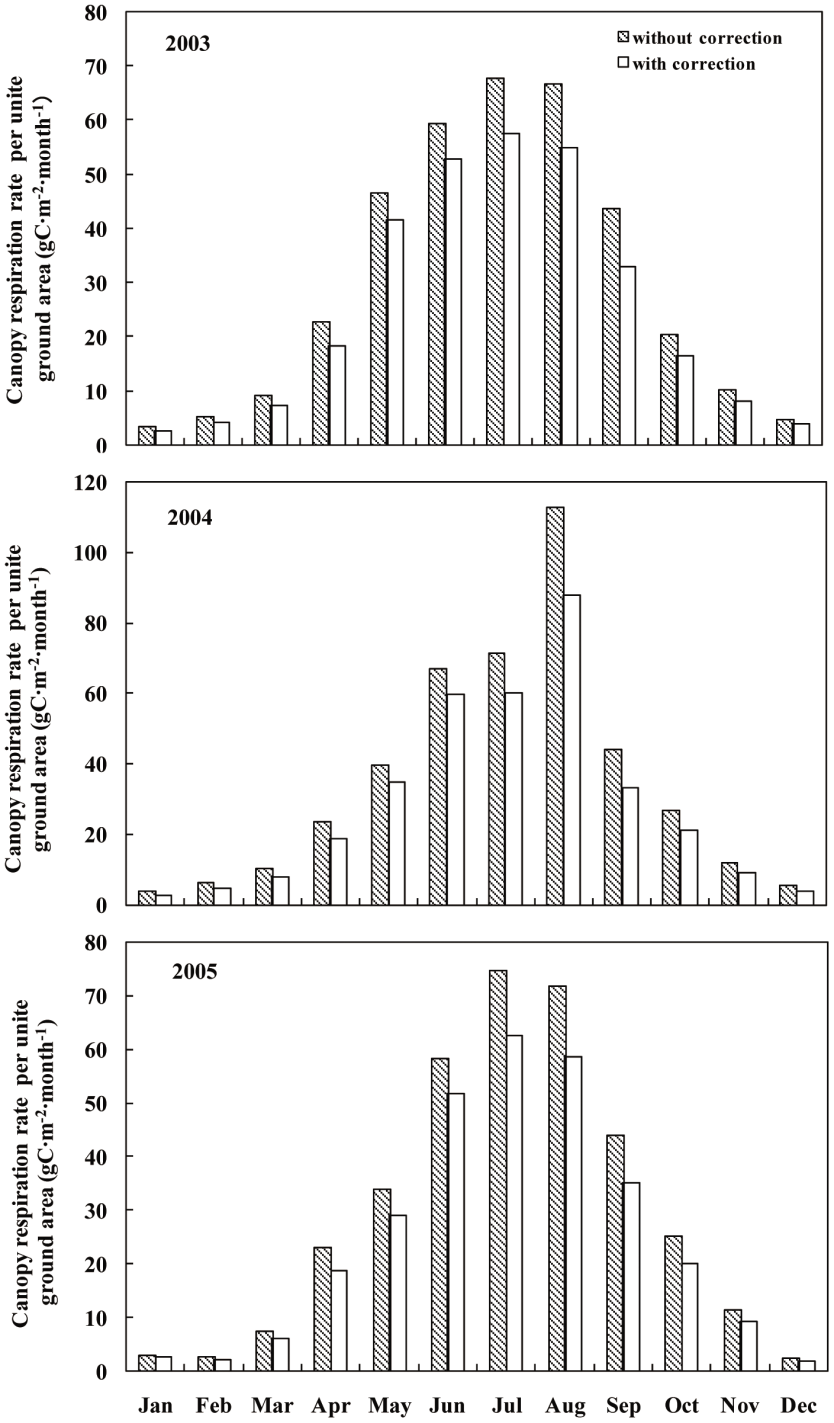
Seasonal trends in monthly canopy respiration rate per unit ground area with and without light inhibition correction from 2003 to 2005.

The effects of seasonal variation on *R*_c_ and *R*_c_^*^ are shown in Fig. 4. Monthly *R*_c_ varied from 2.45 g C·m^−2^·month^−1^ to 112.48 g C·m^−2^·month^−1^ and *R*_c_^*^ ranged from 1.96 g C·m^−2^·day^−1^ to 88.07 g C·m^−2^·day^−1^ from 2003 to 2005. The highest *R*_c_ and *R*_c_^*^ values occurred in July and August, and the lowest in January and December. The discrepancy between *R*_c_ and *R*_c_^*^ reached its highest value in August in all three years. The cumulative values of *R*_c_ and *R*_c_^*^ in each year from 2003 to 2005 are shown in Table 4. Thus, the cumulative values of *R*_c_ were 20.0%, 20.5%, and 20.6% higher than the cumulative *R*_c_^*^ values in 2003, 2004, and 2005, respectively.

**Table 4:** Cumulative annual gross primary production and its components with (indicated by “^*^”) and without light inhibition correction (GPP, gross primary production; *R*_c_, canopy leaf respiration; *R*_st_, stem respiration; *R*_s_, soil respiration; *R*_e_, ecosystem respiration; and NEE, net ecosystem CO_2_ exchange, all units of GPP and its components with and without correction are g C·m^−2^·year^−1^).

Year	*R*_c_	*R*_c_^*^	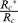	*R*_st_	*R*_s_	NEE	*R*_e_	*R*_e_^*^	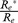	GPP	GPP^*^	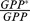
2003	361.08	300.89	83.3%	256.71	593.65	−188.54	1211.44	1151.25	95.0%	1399.98	1339.79	95.7%
2004	376.22	312.25	83.0%	267.47	606.44	−168.08	1250.13	1186.16	95.0%	1418.21	1354.24	95.5%
2005	358.89	297.70	83.0%	255.15	638.99	−185.49	1253.03	1191.84	95.1%	1438.52	1377.33	95.8%

### 3.3 Effect of light inhibition on canopy and ecosystem respiration, and GPP estimation

**Figure 5:**
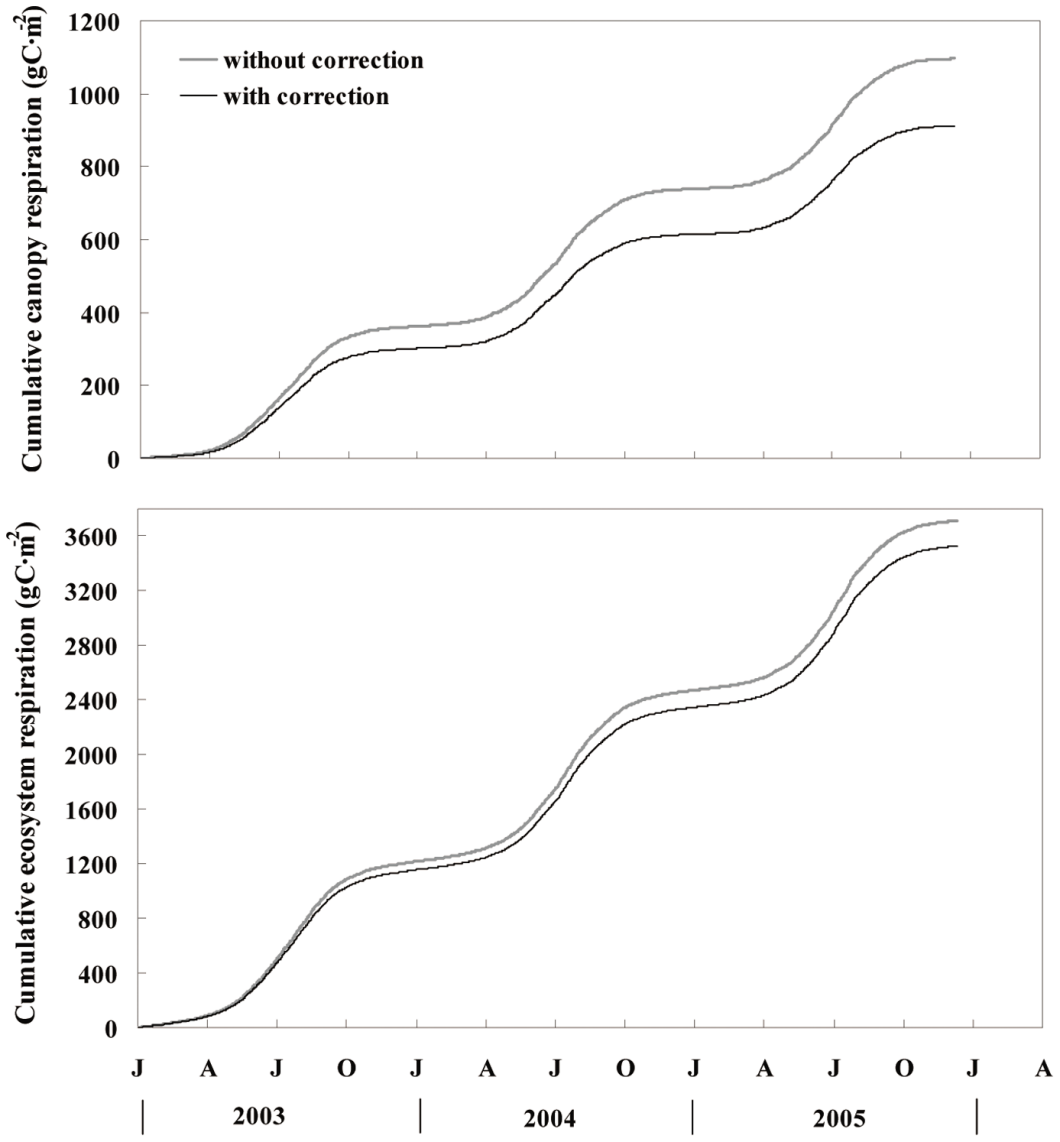
Time course of cumulative canopy respiration (*R*_c_) and ecosystem respiration (*R*_e_) with and without light inhibition correction from 2003 to 2005.

Cumulative values of *R*_c_ and *R*_e_ with and without light-inhibition correction showed similar seasonal variation over the three years (Fig. 5). These values were low in winter and high over the period from June to August in each year. The average difference between the corrected and uncorrected *R*_c_ values is 61.79 g C·m^−2^, which is equivalent to the *R*_e_ overestimation for the three years. The annual sums of components of *R*_e_ (i.e., *R*_c_, *R*_c_^*^, *R*_st_, and *R*_s_), *R*_e_^*^, NEE, and GPP are summarized in Table 4 for the three years. The value of *R*_c_/*R*_e_ range from 28.6% to 30.1%, whereas the value of *R*_c_^*^/*R*_e_^*^ range from by 25.0% to 26.3%, i.e., accounting for daytime canopy respiration inhibition resulted in an *R*_c_/*R*_e_ reduction of 3.7% (Student's *t*-test, P<0.05). The *R*_e_ was 5.2%, 5.4%, and 5.1% higher than *R*_e_^*^, and GPP was 4.5%, 4.7%, and 4.4% higher than GPP^*^ from 2003 to 2005, respectively, owing to daytime canopy light inhibition.

## Discussion

### 4.1 Leaf respiration reduction by light

Leaf-level *R*_L_/*R*_D_ declined exponentially with increasing *Q*, indicating that *R*_L_ is inhibited by light. Although this type of inhibition has been widely reported, the reason remains unclear [8],[34]–[35]. Some studies suggest that light inhibition is merely a phenomenon caused by internal CO_2_ re-fixation [36]–[37]. More studies have shown that leaf dark respiration is clearly inhibited by light [38]–[42]. This has been attributed to increased amounts of ATP and NADPH in response to increasing *Q* levels [38], and the inhibition of respiratory enzymes (pyruvate dehydrogenase and isocitrate dehydrogenase) with increasing ATP and NADPH levels [4],[39]–[42]. In addition, leaf respiration in light was lower than in darkness even if the refixed respiratory CO_2_ was considered [43]. Further, the Krebs cycle, one of main metabolic pathways of leaf dark respiration, was reduced by 95% in light [13]. The mechanism causing light inhibition of respiration is likely related to inhibition of specific enzymes but further investigation of the mechanism is beyond the scope of this work.

### 4.2 Effect of environmental factors on leaf respiration and GPP estimation

Light inhibition of *R*_L_ ranged from 10% to 98% in this study, which reflects *R*_L_ variation in the different species and developmental stages. In addition, *R*_L_ and the effect of light inhibition on this variable vary with environmental factors, for example, light inhibition of *R*_L_ decreased with increasing nitrogen availability [44]–[45], increasing temperature [8],[46], and under elevated CO_2_ [45],[47] and well-watered conditions [48]. These studies indicate that research focusing on the effect of light inhibition on leaf respiration under different environmental conditions has increased in recent years. GPP overestimation was quantified based on the light inhibition of *R*_L_, which was determined by the ecosystem species composition and LAI at certain developmental stages in the current study. Environmental factors such as temperature, precipitation, ambient CO_2_ concentration, and nitrogen deposition were not taken into account in the present study; nevertheless, a basic and important method to quantify the GPP overestimation is provided. Based on this study, the GPP overestimation can be evaluated more accurately if the above-mentioned environmental factors are considered.

### 4.3 Effect of canopy structure on light inhibition and GPP estimation

**Figure 6:**
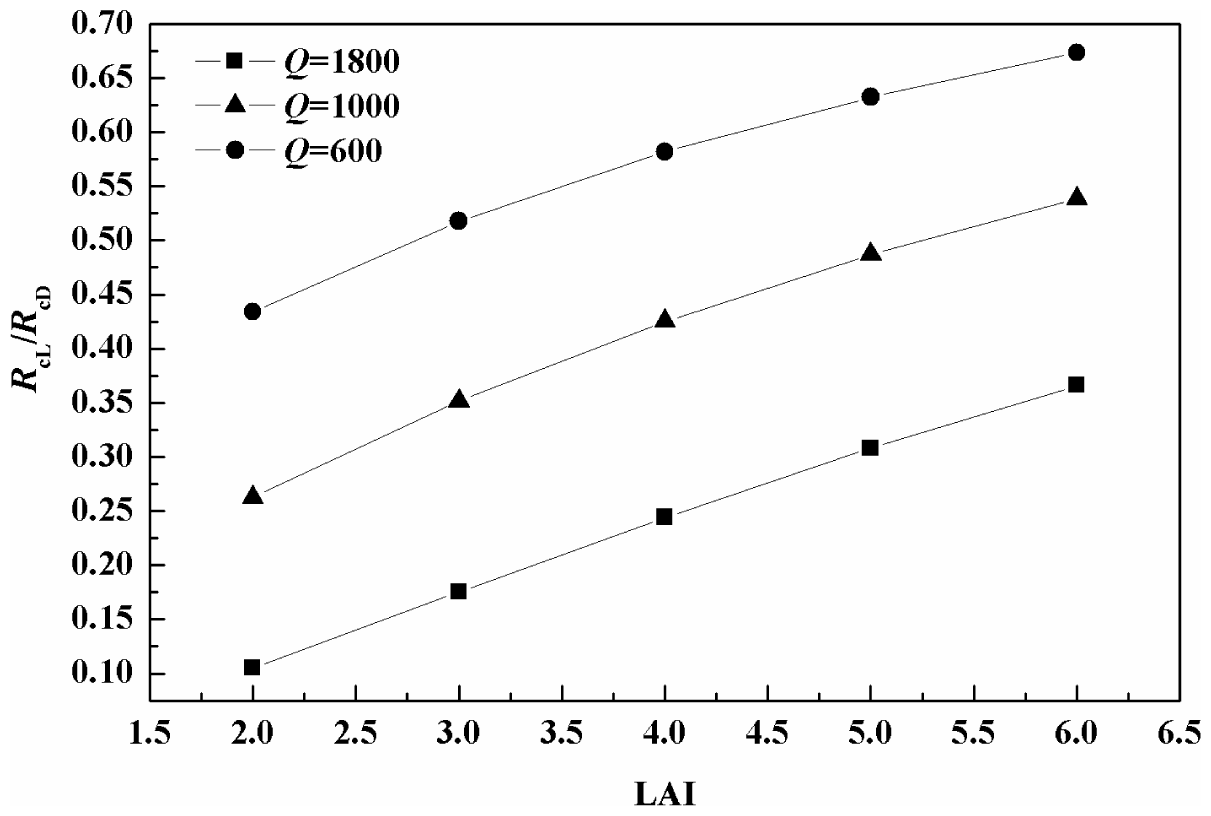
Reduction in canopy-level respiration in light relative to darkness (*R*_cL_/*R*_cD_) as a function of five assumed leaf area indices (LAI) at three photosynthetically active radiation (*Q*) levels.

Leaf area distribution within the canopy changes as forests grow and develop. GPP overestimation in this study (4.6%) was lower than that (>20%) in an old beech forest ecosystem [16]. The discrepancy may result from the in-canopy *Q* value, which was affected by LAI. In this study, the entire canopy was divided into multiple layers, which avoided the overestimation of light on leaves and consequently, the decrease in *R*_c_ also avoided the overestimation. In a study conducted by Bruhn *et al.* (2011), the *Q* value above the canopy was used to represent the value of the entire canopy, ignoring light attenuation in the canopy [13]. The *R*_cL_/*R*_cD_ value increased with increasing LAI at each *Q* level (Fig. 6). The results indicate that LAI is an important factor that affects canopy-level respiration reduction in light. In comparison, small reductions in *R*_c_ may occur in ecosystems characterized by high LAI values as a result of the self-shading effect on *Q*.

**Figure 7:**
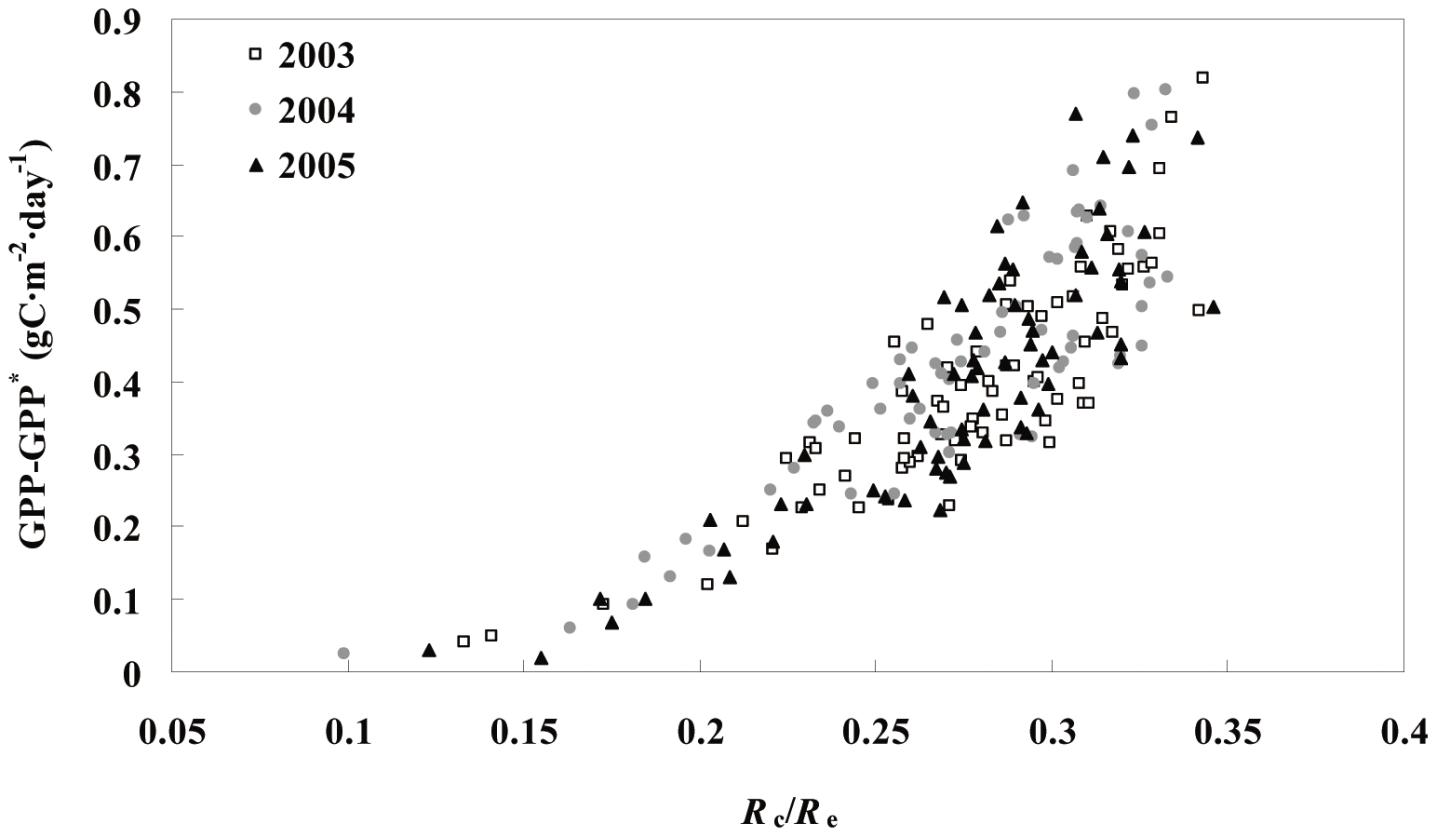
GPP reduction (GPP–GPP^*^) calculated as a function of the ratio of daily canopy respiration to ecosystem respiration (*R*_c_/*R*_e_).

In addition, canopy structure impacts the canopy respiration ratio (as a component of ecosystem respiration), which may affect GPP overestimation. Model simulation results suggest that GPP overestimation in our study was approximately 4.6%. The GPP overestimation value reported by Wohlfahrt *et al.* (2005) was 11–13% [17] under a high light inhibition scenario which is similar to the light inhibition used in our study. In the present study, the forest ecosystem *R*_c_/*R*_e_ was 29%, compared with 42% in a mountain meadow ecosystem [17]. Differences in the *R*_c_/*R*_e_ values may result in the discrepancy in GPP overestimation between these two ecosystems. The variation in the discrepancy between GPP and GPP^*^ increased exponentially with increasing *R*_c_/*R*_e_ values (Fig. 7). A greater amount of leaf respiration will be ignored if the effect of light inhibition is not taken into account in ecosystems with a high *R*_c_/*R*_e_ value. Based on the above discussion, large reductions in GPP may be expected in ecosystems with high proportions of canopy respiration relative to ecosystem respiration, such as a Finland agricultural ecosystem where the *R*_c_/*R*_e_ value reached 50% [49]. In contrast, there is a lower GPP reduction in ecosystems with lower *R*_c_/*R*_e_ values, such as a mixed temperate forest with an *R*_c_/*R*_e_ value of 31% to 33% [30],[50], and other forest ecosystems where *R*_c_/*R*_e_ was less than 31% [11],[51]–[53].

### 4.4 Impact of leaf respiration differences between seedlings and trees on GPP estimation

The measurements of mature trees are usually conducted on cut branches because of the difficulty associated with direct measurements. In this study, leaf respiration of seedlings were used as a proxy for leaf respiration of trees because of the long duration required when estimating leaf respiration in light when using the Laisk method. It was not feasible to measure leaf dark respiration in vitro in mature trees because detached leaves do not maintain their physiological activity because the water supply is disrupted. However, numerous studies have indicated that many biological processes change with increases in tree age [54]–[56]. Leaf respiration and the effect of light inhibition on leaf respiration may differ between seedlings and mature trees. The accuracy of results may be affected should this difference be ignored. Therefore, differences in leaf respiration characteristics between seedlings and trees should be analyzed in future studies.

## Conclusions

Increasing attention is being given to understand light inhibition on leaf respiration, but few studies have quantified the impact of light inhibition on GPP estimation. Our results demonstrated that inhibition of leaf respiration during the day is species-specific. Light inhibition of leaf respiration increased exponentially with increasing light intensity for the dominant tree species of a mixed mature temperate forest in northeast China. Canopy respiration and GPP were overestimated across the three years of the study by approximately 20.4% and 4.6%, respectively, when leaf respiration reduction in light was ignored. It is important that leaf respiration reduction by light is taken into account when estimating the GPP of the ecosystem with high LAI. In the present study, numerous factors that may influence leaf respiration reduction were considered to provide accurate GPP estimates. Therefore, this study provides important methodological approaches that can be applied to other ecosystems with different species. Collectively, these results are vital to make predictions about how leaf respiration reduction by light will impact ecosystem carbon measurements.

## Supporting Information

Table S1 **Nomenclature.**
(DOCX)

Appendix S1 **A brief description of the model of solar radiation transmission through the canopy.**
(DOCX)
