# Supplementary material for: Estimating Daytime Ecosystem Respiration to Improve Estimates of Gross Primary Production of a Temperate Forest
Source: PLoS One. 2014 Nov 24;9(11):e113512. doi: 10.1371/journal.pone.0113512 (PMC4242619; doi:10.1371/journal.pone.0113512)
Supplement: Table S1 — Nomenclature. (DOCX) [file pone.0113512.s001.docx]

**Table S1. Nomenclature.**

| **Nomenclature** | |
| --- | --- |
| *Q* | photosynthetically active radiation (μmol∙m^−2^∙s^−1^) |
| LAI | leaf area index (m^2^∙m^−2^) |
| *R*_L_ | leaf respiration in light (μmol∙m^-2^∙s^-1^, g C∙m^-2^∙day^-1^) |
| *R*_D_ | leaf respiration in darkness (μmol∙m^-2^∙s^-1^, g C∙m^-2^∙day^-1^) |
| *R*_cL_ | canopy respiration in light (μmol∙m^-2^∙s^-1^, g C∙m^-2^∙day^-1^) |
| *R*_cD_ | canopy respiration in darkness (μmol∙m^-2^∙s^-1^, g C∙m^-2^∙day^-1^) |
| *R*_c_ | canopy respiration (μmol∙m^-2^∙s^-1^, g C∙m^-2^∙day^-1^) |
| *R*_s_ | soil respiration (μmol∙m^-2^∙s^-1^, g C∙m^-2^∙day^-1^) |
| *R*_st_ | stem respiration (μmol∙m^-2^∙s^-1^, g C∙m^-2^∙day^-1^) |
| *R*_c_^*^ | canopy respiration corrected for reduction of leaf dark respiration in light (μmol∙m^-2^∙s^-1^, g C∙m^-2^∙day^-1^) |
| *R*_e_ | ecosystem respiration (μmol∙m^-2^∙s^-1^, g C∙m^-2^∙day^-1^) |
| *R*_e_^*^ | ecosystem respiration corrected for reduction of leaf dark respiration in light (μmol∙m^-2^∙s^-1^, gC∙m^-2^∙day^-1^) |
| GPP | gross primary production (g C∙m^-2^) |
| GPP^*^ | gross primary production corrected for reduction of leaf dark respiration in light (g C∙m^-2^) |
