# Supplementary material for: Estimating Daytime Ecosystem Respiration to Improve Estimates of Gross Primary Production of a Temperate Forest
Source: PLoS One. 2014 Nov 24;9(11):e113512. doi: 10.1371/journal.pone.0113512 (PMC4242619; doi:10.1371/journal.pone.0113512)
Supplement: Appendix S1 — A brief description of the model of solar radiation transmission through the canopy. (DOCX) [file pone.0113512.s002.docx]

**Appendix S1.** **A brief description of the model of solar radiation transmission through the canopy.**

Solar radiation (*S*_0_) was separated into diffuse (*S*_d0_) and direct beam components (*S*_b0_) as described in Leuning *et al*. (1995) [31]:

$S_{d0}=f_{d}\cdot S_{0}$ (1)

$S_{b0}=(1-f_{d})\cdot S{}_{0}$ (2)

where *f*_d_ is the fraction of diffuse radiation. *S*_0_ was observed, and *f*_d_ was estimated using a segmented function of atmospheric transmissivity (*τ_a_*) [57]:

$f_{d}=\left\{\begin{matrix} 1 & for & \tau_{a}<0.3 \\ 1-2\left(\tau_{a}-0.3\right) & for & 0.3\leq\tau_{a}\leq0.7 \\ 0.2 & for & \tau_{a}>0.7 \end{matrix}\right.$ (3)

where *τ_a_* was estimated using;

$\tau_{a}=S_{0}/\left(S_{c}\cdot\sin\beta\right)$ (4)

$S_{c}=1367\left\{1+0.033\cos\left[2\pi\left(t_{d}-10\right)/365\right]\right\}$ (5)

where *t_d_* is Julian day; *S*_c_ is solar radiation; *β* is solar altitude (°) and sin*β* can be calculated as:

$\sin\beta=a+b\left[2\pi(t_{h}-12)/24\right]$ (6)

where *t_h_* is solar time, and the *a* and *b* parameters can be calculated using functions 7 to 11:

$a=\sin\lambda_{l}\cdot\sin\delta_{s}$ (7)

$b=\cos\lambda_{l}\cdot\cos\delta_{s}$ (8)

$\sin\delta_{s}=-\sin(23.5\pi/180)\cdot\cos\left[2\pi(t_{d}+10)/365\right]$ (9)

where *λ_l_* is latitude and *δ_s_* is solar declination.

The *Q* varied with canopy location; therefore, the *Q* of each canopy layer was calculated separately.

$Q_{d0}=f_{d}\cdot Q_{0}$ (10)

$Q_{b0}=\left(1-f_{d}\right)\cdot Q_{0}$ (11)

where *Q_d0_* and *Q_b0_* represent the diffusion and direct radiation components of the observed *Q*_0_, respectively, and 1 W∙m^-2^ (*S_0_*) =2 μmol∙m^-2^ s^-1^ (*Q*_0_) [31]. Shaded leaves receive diffuse radiation only, and sunlit leaves receive diffuse and direct beam radiation. The *Q* absorbed by shaded (*Q_sh_*) and sunlit leaves (*Q_sl_*) was calculated as:

$Q_{sh}(\xi)=Q_{ld}'(\xi)+Q_{lbs}(\xi)$ (12)

$Q_{sl}(\xi)=Q_{lb}(\xi)+Q_{sh}(\xi)$ (13)

In equation (12), *ξ* is cumulative leaf area index from the top canopy; *Q_ld_′* and *Q_lbs_* represent the incident diffuse and scattered beam radiation, respectively, which are related to the extinction coefficient for radiation and the leaf area index (LAI) [58-59]. In equation (13), *Q_lb_* represents absorbed beam radiation [31]. *Q_ld_′* and *Q_lbs_* were calculated as:

$Q_{ld}'(\xi)=Q_{d0}\cdot k_{d}'\cdot(1-\rho_{cd})\cdot\exp(-k_{d}'\cdot\xi)$ (14)

$Q_{lbs}(\xi)=Q_{b0}\left[k_{b}'\cdot(1-\rho_{cb})\cdot\exp(-k_{b}'\cdot\xi)-k_{b}\cdot(1-\sigma_{l})\cdot\exp(-k_{b}\cdot\xi)\right]$ (15)

where *ρ_cb_* is the canopy reflection coefficient for direct radiation, which can be calculated with the canopy reflection coefficient for horizontal leaves (*ρ_h_*) using equation (16) [58]. *ρ_cd_* is the canopy reflection coefficient for diffusion radiation, with values of 0.057 and 0.389 for visible and near-infrared light, respectively, when diffusion radiation is uniformly distributed in a canopy with spherical leaf angle distribution [31, 59]. *k_d_*′ and *k_b_*′ represent the canopy extinction coefficient of diffusion and direct beam radiation, respectively, calculated using *k_b_* and *k_d_* in equations (17)-(18):

$\rho_{cb}=1-\exp\left[\left(-2\rho_{h}\cdot k_{b}\right)/\left(1+k_{b}\right)\right]$ (16)

$k_{d}'=k_{d}\cdot\left(1-\sigma_{l}\right)^{1/2}$ (17)

$k_{b}'=k_{b}\cdot\left(1-\sigma_{l}\right)^{1/2}$ (18)

In equation (16), *ρ_h_* can be calculated as:

$\rho_{h}=\frac{1-\left(1-\sigma_{l}\right)^{1/2}}{1+\left(1-\sigma_{l}\right)^{1/2}}$ (19)

In equations (17)-(18), *k_b_* and *k_d_* represent the extinction coefficients of direct beam and diffusion radiation, respectively, in an ideal canopy where a leaf is viewed as a “black body”. *σ_l_* is the scattering coefficient (*σ_l_*= 0.2 for visible light; *σ_l_*= 0.8 for near-infrared radiation). Goudriaan and Van Laar (1994) reported that *k_d_* is approximately 0.8 for spherical leaf angle distributions [59]. *k_b_* is a function of *β* as follows:

$k_{b}=0.5/\sin\beta$ (20)
